# Women’s knowledge about the conditional cash incentive program and its association with institutional delivery in Nepal

**DOI:** 10.1371/journal.pone.0199230

**Published:** 2018-06-21

**Authors:** Shanta Pandey

**Affiliations:** Boston College School of Social Work, Chestnut Hill, Massachusetts, United States of America; The Ohio State University, UNITED STATES

## Abstract

**Background and purpose:**

Home deliveries increase the risk of maternal and child mortality. To increase institutional deliveries, South Asian countries have introduced various forms of Conditional Cash Transfer (CCT) schemes that offer women cash if they come to deliver at a health facility. In 2005, Nepal introduced its *Safe Delivery Incentive Programme* (SDIP)—a cash incentive program nationwide to boost the rate of institutional delivery and care from health professionals at childbirth. This study asks the following research questions: How informed were Nepalese women about the cash incentive program? Does knowledge about the cash incentive program correlate with institutional delivery?

**Methods:**

Data to answer these questions come from the 2011 Nepal Demographic and Health Survey (NDHS). This is a nationally representative data collected from 12,674 women between 15 and 49 years of age, of which 4,036 had given births in the past five years. Multiple logistic regression was employed to predict if knowledge about the cash incentive program increased the odds of institutional delivery controlling for sociodemographic and geographic factors.

**Results:**

Approximately 90% of the women knew about the SDIP. About 42% of the women who knew about the SDIP and 13% of the women who did not know about the SDIP had their most recent delivery at a health institution. The odds of institutional delivery increased nearly three-fold (OR = 2.70; CI: 1.59–4.59) among women who knew about the SDIP compared to women who did not know about the SDIP. Other factors that predicted institutional delivery included education, wealth, urban status, first birth, the number of antenatal care visits, and exposure to news media.

**Conclusion:**

This study shows that there is a correlation between women’s knowledge about the SDIP and increased institutional delivery. Nepal’s health and social work professionals should inform all women of reproductive age about the program so that they can make more informed delivery decisions.

## Introduction

Globally, about 5.9 million children die annually within the first five years of birth and these deaths are more concentrated during the earliest days of their lives: one million children die on the day of birth, two million die within the first week of birth, and 2.7 million die within the first four weeks of birth or neonatal period [1]. Over the years, child deaths between ages two and five have declined, but the neonatal deaths have remained persistently high. Three causes—pre-term births, infections, and asphyxia—contribute to over 80% of neonatal mortality[2]. These deaths could be eliminated if the delivery occurred in a health facility where informed birth attendants attend to pregnant women and newborn babies with complications. In South Asia, however, women have traditionally chosen the home delivery option over institutional delivery with a reduced risk of maternal and child mortality. To reduce child and maternal mortality rates, nearly all developing countries have increased the supply of public health facilities and the number of health workers. The conventional supply-side approaches, however, have not increased service use among the poor especially when such services are expensive and unaffordable [3]. To make obstetric care affordable, in 2005 India introduced *Janani Suraksha Yojana (JSY)*, a Conditional Cash Transfer (CCT) program that offers poor women up to $30 in cash (equivalent to 3 weeks of salary) if they deliver at a health facility [4]. In 2005, Nepal also introduced its *Safe Delivery Incentive Programme* (SDIP)—a nationwide program that offers cash to women who deliver at a health facility and to health care providers who attend delivery [5–7]. Evaluations of the SDIP are mostly localized and have assessed administrative and implementation capacities of national, district, and local health institutions [8–10]. None have examined the program’s nation-wide impact on institutional delivery. This study is a national analysis of how knowledgeable were Nepalese women about the SDIP? And, did the knowledge of the SDIP correlate with institutional delivery?

## Background

In countries with high rates of maternal and child mortalities, the use of maternal and child health services is low. Nepal’s maternal mortality ratio at 258/100,000 live births is one of the highest in the world [11]. In 2013–14, approximately 45% of women in Nepal, 63% in Bangladesh, and 21% in India gave their most recent birth at home [11]. Prior researchers have indicated that many poor households avoid institutional care because of the high cost [12]. Theoretically, all institutional deliveries in Nepal are free, but there are hidden costs such as prescription drugs, food, drink, clothes, transportation, and lost wages of accompanying family members–that in total can range from about US $243 for a normal delivery to US $322 for a Caesarian Section delivery [7]. One study estimated that the average cost of hospital delivery for a poor household in Nepal was equivalent to three months of household income [12]. Moreover, many families do not believe that it is unsafe to deliver at home. Most women in Bangladesh (77%) and India (79%) who delivered at home indicated that hospital delivery was neither customary nor necessary [13, 14]. Given the prevailing custom of home delivery, the decision to spend a significant portion of the household income on hospital delivery would be extraordinary and burdensome.

Conditional cash transfer (CCT) programs to improve health and education became popular in Latin America since Mexico introduced its CCT, *PROGRESSA* in 1997 [15]. Other countries in Africa and Asia including Nepal [16], India [17], and Bangladesh [18] have adopted their versions of the CCT programs. In Latin American countries, these programs are means tested. Cash is regularly transferred (bimonthly, monthly) to poor women with young children, school-aged children, and pregnant women if they meet the program requirements: use preventive health services, attend health education sessions, and ensure school enrollment of children. The CCT programs that transfer cash directly to women in Latin American countries have improved children’s health, nutrition and education [19, 20]. They have also improved women’s financial security, self-esteem, social status, ability to make financial decisions and access to health care services [19, 21]. In Bangladesh, their pilot voucher program significantly increased the use of antenatal, delivery, and postnatal care with qualified providers [18].

### SDIP: Nepal’s cash incentives program

In 2005, Nepal introduced the SDIP. Since 2009, it is also known as *“Aama (or mother) Program*.*”* The program is being advertised through Nepal’s public media (radio, television, newspapers) and health care providers in hospitals, primary health centers, and health posts. At the Village Development Committee (VDC) level, the primary health centers and health posts are staffed by general physicians, nurses, health assistants, and Auxiliary Nurse Midwives (ANMs). ANMs have midwifery training and are employed in community health clinics to increase rural women’s access to reproductive health and maternal and child health. Also, every VDC has one (sometimes more than one) female community health volunteer (FCHV) who attends to local women’s health needs (e.g., prenatal care, family planning, child immunization, and nutrition) [22]. These FCHV attend the meetings of mothers’ group for health, that focus on the health of pregnant and breastfeeding women [23]. These maternity care providers are also responsible for promoting information about the cash incentives program. Pregnant women who use antenatal services may also receive information about the SDIP from their health care providers. Hence, it is likely that the women who use more antenatal care visits receive information about the SDIP multiple times whereas women who do not or seldom use antenatal care may hear much less about the program. It is also possible that women learn about the cash incentives from their friends, neighbors, and relatives.

The SDIP or the cash incentives program includes cash payments by the government of Nepal through the health institutions—hospital or PHC—to service users and service providers. The program defrays some of the patient’s cost of transportation to health institutions. Service providers also receive cash to cover some of the costs associated with delivery, prescription drugs, and medical equipment. Overall, the goal of SDIP is to create demand-side incentives, increase institutional delivery and reduce birth-related maternal and child deaths. More specifically, the program has the following components [5, 24, 25]:

- Nepal has the universal cash incentives to women covering all 75 districts, but the amount varies by geography. For each institutional delivery, patients in the northern mountain districts of Nepal receive NRS 1,500 (approximately US $15), those in the middle hill districts including Kathmandu receive NRS 1,000 ($10), and those from the southern Terai Districts of Nepal receive NRS 500($5).
- Nepal also has the incentives for service providers for attending a delivery. Service providers receive NRS 300 ($3) per delivery attended at an institution and NRS 200 ($2) per delivery for assisting delivery at home.
- Finally, Nepal provides a subsidy for small health institutions in remote and mountainous districts with up to 25 beds. They receive NRS 1,000 ($10), NRS 3,000 ($3) and NRS 7,000 ($7) respectively for normal, complicated, and Caesarean Section deliveries.

Initially, the incentive was restricted to women delivering at a public health facility, for up to two children or complicated pregnancies. Women who requested private wards/cabins were ineligible for the SDIP [5, 25]. In November 2007, Nepal removed the restriction of “up to two children” and made any mother delivering in a health facility, regardless of the number of children as eligible for the SDIP [24]. Women who request private wards/cabins, however, continue to be ineligible for the incentive and must pay for services [5, 25]. Also in 2007, Nepal prioritized the allocation of funds to reach poor women in remote districts. First priority was to provide incentives to mothers; second priority was to provide institutional subsidy to government heath institutions in Nepal’s 25 marginalized districts; third priority was to provide incentives for health workers engaged in institutional delivery; and fourth, if funds were available, health workers engaged in-home delivery would also receive a cash incentive [24]. Local health institutions distributed the cash to mothers who came to deliver at a health facility and to service providers attending the delivery, either at home or in the facility [6].

### CCT programs’ impact on maternal use of health services

The impact of cash incentive programs on the maternal use of antenatal and delivery care has been encouraging. In Mexico the average number of antenatal care visits increased among young women living in areas with more exposure to *Oportunidades* (*PROGRESSA*) when compared to disadvantaged, indigenous women had less access to skilled delivery care [26]. This study recommends developing and implementing strategies to increase awareness about CCT program among marginalized women in Mexico. India’s CCT program—*Janani Suraksha Yojana (JSY)*)—has increased institutional delivery and reduced perinatal and neonatal deaths [17, 27–30], but like in Mexico, the poorest and least educated women were least likely to receive the JSY payments [17, 31]. In Nepal, institutional deliveries have slowly risen since the launch of the SDIP; but many women did not know about the program and not all those who knew the program used it [16, 25]. One reason for the underuse of SDIP was because service providers were often unavailable at local facilities and women either went to private facilities or delivered at home. Other studies noted bureaucratic inefficiencies in implementing SDIP at all levels [6, 32]. As in Mexico and India, it has been observed that SDIP recipients in Nepal were disproportionately from affluent households [33].

In this paper, women’s delivery decisions are framed from the perspective of diffusion of innovation and social learning theories. Diffusion of innovation theory (DIT), proposed by Everett Rogers, postulates that adoption of a new idea, behavior, or a technology depends upon knowledge about that innovation and its perceived attributes including its relative advantage in terms of costs and benefits and the degree to which it is socially compatible and perceived as being superior to the one it replaces [34–36]. To generate demand for institutional delivery through cash incentives, the women should be knowledgeable about the SDIP and that institutional delivery should be affordable, socially acceptable, and produce better health outcomes[36]. Similarly, Bandura’s, social learning theory (SLT) has been used to explain a wide range of human behaviors [37–43]. He proposed that social learning and change in behavior, including the use of antenatal care and institutional delivery occur within a social context; people learn from one another by observing, imitating and modeling. If family, friends and community members have a positive experience and endorse changes in personal behavior, such changes have a higher likelihood of becoming habits [44, 45]. Most women in developed nations choose institutional delivery. Studies from Nepal show that women from lower economic status, residing in rural areas, married in teen years, with low education, with multiple pregnancies, and with limited access to antenatal care were more likely to choose home delivery[14, 46–49]. In the current study, it is reasonable to anticipate that if a woman had a positive experience of delivering at a health facility—regarding delivery, child survival, and the receipt of cash incentive—she and her family would inform their friends and neighbors and that would have a ripple effect in increasing the rate of institutional delivery. Informed by these theories, the current study asks the following two questions: How informed were Nepalese women about the SDIP? Did the knowledge about the SDIP translate into an institutional delivery controlling for other known social, economic, and demographic factors?

## Methodology

The current study used the *2011 Nepal Demographic and Health Survey (NDHS)* data, a nationally representative comprehensive survey, which used a two-stage, stratified sample design to collect data from 10,826 households which resulted in a completed interview from 12,674 women between 15 and 49 years of age (for details see, [50]). The *2011 NDHS* asked if women delivered in the past five years, which coincided with the implementation of Nepal’s SDIP. Of the total sample, 4,036 women had given birth in the past five years. This study analyzed these women’s exposure to the SDIP and the place of delivery of their most recent birth.

### Ethics statement

This study is based on secondary analysis of existing, nationally representative, publicly available *Demographic and Health Surveys* data. I downloaded the *2011 NDHS* data from https://dhsprogram.com/data/available-datasets.cfm with permission from the DHS website. As the data had been anonymized and were available in the public domain, approval from an Institutional Review Board was not sought for this analysis. Data analysis was conducted using SAS version 9.2.

### Measures

#### Dependent variable

The outcome variable is women’s choice for the place of delivery. Women who had given birth in the past five years were asked the place of delivery: home or health institution—government, nongovernment or private institution. Women who delivered their youngest child at a health institution—government, nongovernment or private institution—were coded as 1, and those who gave birth at own home or in another home were coded as 0.

#### Independent variable

Women’s awareness about the SDIP program is the independent variable of interest. The NDHS survey asked women who had given births in the past five years the following question: *Does a woman get a cash incentive if she delivers her baby at a government health facility*? The answer category included: yes, no and don’t know. Those who decisively answered “yes” were coded as 1 and those who said no and don’t know as 0.

#### Control variables

In the logistic regression, before assessing the correlation between women’s knowledge about the cash incentives and the outcome variable, I controlled for their demographic characteristics *(age at marriage and interview*, *number of children*, *education*, *their husband’s education*, *household wealth*, *religion*, *region of residence)*, barriers to use health services *(cost of care and distance to health facility)*, *total number of antenatal care visits for their most recent live birth*, their extent of *exposure to news media (newspaper*, *radio and television)* and their *exposure to maternal health education programs* broadcasted through radio and television (see Table 1 for operational definition of all the covariates).

**Table 1:** Operational definitions of co-variates used in the current analysis.

Variables	Operationalized descriptions
**Age at interview**	A continuous variable representing women’s age at the time of interview.
**Age at marriage**	This continuous variable representing women’s age at first marriage was dummy coded. If women were married at or after age 20 which is the legal age for marriage in Nepal, they were coded as 1; whereas if they were married before age 20, they were coded as 0.
**Number of children**	*Number of children* women had given birth were coded into three dummy variables: Women with one child, women with two children, and those with more than 2 children.
**Education: Women and Husband’s**	Women’s *(*and their husband’s*) education* included three dummy variables: no education, primary education (that included pre-primary to the completion of 5^th^ grade), secondary and beyond education (6^th^ grade and above).
**Wealth index**	Using a long list of variables that assessed household's ownership of assets (such as radio, televisions, bicycles, mobile phone, refrigerator, table, chair, bed, sofa, and so on), the DHS data classified households into 5 wealth indices: poorest, poorer, middle, richer and richest. This variable was recoded into three dummy variables—*poor*, *middle and rich class*. *Poor class* included poorest and poorer, *middle class* remained unchanged, and *rich* included richer and the richest.
**Religion**	Hindu women were coded as 1; else they were coded as 0.
**Rural/Urban**	Urban residence = 1; rural residence = 0
**Ecological region of their residence**	Three geographic regions of Nepal, the northern Mountain region, middle hills, and southern Terai. These three variables were dummy variables as *Mountain*, *Hill and Terai*.
**Barriers use health services:** Problem w/Money Problem w/Distance	Respondents were asked: Many different factors can prevent women from getting medical advice or treatment for themselves. When you are sick and want to get medical advice or treatment, is each of the following a big problem or not? The sub-questions included: *Getting money needed for advice or treatment*? *The distance to the health facility*? Answer categories for each question included: big problem (1) and not a big problem (2). For each response if the answer was a big problem it was coded as 1; else = 0.
**Total # of antenatal care visits for the most recent live birth**	Women’s response to total the number of antenatal care visits used for their most recent live birth was dummy coded into three variables: no visit, 1 to 3 visits, and 4 or more visits.
**Women’s exposure to news media index**	Three items were used: (a) Do you read a newspaper or magazine, at least once a week, less than once a week or not at all? (b) Do you listen to the radio, at least once a week, less than once a week or not at all? (c) Do you watch television, at least once a week, less than once a week or not at all? Response included: at least once a week (= 2); less than once a Week (= 1); and not at all (= 0). An index was created whose values ranged from 0 to 6 where 0 means no media exposure and 6 means weekly exposure to all 3—newspaper, radio and tv.
**Women’s exposure to health education programs**	The survey asked: In the last few months have you heard or seen the following programs on the radio and/or television: (a) Jana Swastha Radio Karyakram? (b) Janasankhya Chetana ka Sworeharu Radio Karyakram? (c) Hamro Swastha Radio Karyakram? (d) Ama radio Karyakram? (e) Hamro Swastha TV Karyakram? (f) Jeevan Chakra TV Karyakram? (g) Thorai bhaye pugi sari TV Karyakram? (h) Ama TV Karyakram? (i) Sathi Sanga Manka Kura Radio Karyakram? (j) Jeevan Jyoti Radio Karyakram? The response categories for each question included yes = 1 and no = 0. Those who had heard/seen any one of these programs were coded 1; else = 0.

### Statistical analyses

The dependent variable was binomial. Thus, weighted (probability weight) descriptive and logistic regression (*survey logistic*) analyses are employed using the SAS version 9.2 that incorporates complex sample designs to assess the relationships between institutional delivery and the independent variable controlling for other factors. Of the 4,036 women who had given birth in the past five years, the logistic regression model is based on 3,997 cases. A total of 39 cases (or less than 1% of the sample) were excluded due to missing values. Twenty-four women did not answer their place of delivery and additional 15 women did not answer their husband’s educational level.

### Diagnostic tests

Before running the logistic regression model, I checked for multicollinearity among the independent and control variables. The highest variance inflation factor (VIF) for variables in the logistic regression model was 2.82 and the lowest tolerance value was 0.35, indicating lack of any serious problem of multicollinearity [51]. To assess the fitted model’s overall departure from the observed data, the Hosmer and Lemeshow goodness-of-fit test was evaluated using the unweighted data. If the difference between the observed and predicted frequencies was high then obtained *Chi-square* value would be high and the null hypothesis of a close fit between observed and predicted probabilities would be rejected. Thus, an insignificant *Chi-square* value implies that the model fits the data. Also, tests to assess the predictive ability of the model were conducted. The results are reported under the goodness-of-fit test.

## Results

Of the women who had delivered in the past five years, almost 90% knew about the SDIP. However, only 39% delivered at a health facility. One in two women had made four or more antenatal visits to health care providers during their last pregnancy, the WHO recommended number of visits for a normal pregnancy (see Table 2). A higher proportion of the women who knew about the SDIP delivered at a health facility (42%) compared to those who were unaware of this program (13%). Also, 54% of the women who knew about the SDIP had made four or more antenatal care visits, compared to 20% of the women who did not know about the program.

**Table 2:** Weighted descriptive results using individual data from Nepal, 2011.

Variables	All women who had delivered in past 5 yrs (N = 4,036)		Women who knew about cash incentive (N = 3,716)		Women who did not know about cash incentive (N = 320)	
	%		%		%	
**Place of delivery of youngest child**						
Health institution (public or private)	39.18		42.27		12.54	
Home	60.82		57.73		87.46	
**# of antenatal care visit during last pregnancy**						
0 visit	15.10		12.60		36.73	
1 to 3 visits	34.67		33.74		42.79	
4 or more visits	50.22		53.66		20.48	
**Women’s education**						
No education	43.98		41.80		62.74	
Primary (1-5^th^ grade)	20.04		19.74		22.62	
Secondary & beyond (6^th^ grade & more)	35.98		38.45		14.64	
**Husband’s education**						
No education	21.10		19.60		34.24	
Primary (1-5^th^ grade)	23.79		22.87		31.89	
Secondary & above (6^th^ grade & above)	55.11		57.53		33.87	
**Religion**						
Hindu	82.91		83.65		76.50	
Other	17.09		16.35		23.50	
**Wealth index**						
Poor	45.20		43.04		63.86	
Middle	20.99		21.36		17.86	
Rich	33.81		35.61		18.28	
**Rural/urban residence**						
Urban	10.14		10.58		6.37	
Rural	89.86		89.42		93.63	
**Ecological Region**						
Hill	40.07		38.99		49.38	
Mountain	07.37		7.88		2.99	
Terai	52.56		53.13		47.63	
**Problem with money**	52.69		50.43		72.24	
**Problem with distance**	53.94		52.05		70.26	
**Exposed to health education programs**	43.59		46.46		18.85	
**Number of children**						
One child	31.27		32.70		18.93	
Two children	28.12		28.64		23.70	
More than 2 children	40.60		38.66		57.37	
**Married at or after age 20**	22.05		22.54		17.80	
**Knows about Cash Incentives**	89.63		100		0	
*Continuous variables*	Mean	std	Mean	std	Mean	Std.
**Total pregnancies**	2.88	2.01	2.79	1.91	3.64	2.75
**Total children**	2.63	1.82	2.54	1.74	3.39	2.44
**Age at 1**^**st**^ **marriage**	17.43	3.16	17.47	3.14	17.10	3.37
**Age at interview**	26.96	6.15	26.72	5.88	29.03	8.33
**Exposure to news media index**	2.49	1.64	2.59	1.62	1.62	1.54

To understand the correlation between knowledge about the cash incentive with institutional delivery, a multiple logistic regression analysis was performed (See Table 3). The model controlled for the demographic characteristics, antenatal care use, barriers to health service use, the extent of exposure to news media, and exposure to health education programs, and then tested the effect of knowledge about the SDIP on institutional delivery. Controlling for other factors, those with knowledge about the cash incentives were nearly three times as likely to deliver at a health facility as those without knowledge (OR = 2.70; CI: 1.59–4.59). Among the control variables, use of antenatal care significantly increased the odds of institutional delivery. Women who made one to three visits during their pregnancy were twice as likely to deliver at a health facility compared to women who never made any visits (OR: 2.01; CI: 1.25–3.24). Women who made at least four visits, as recommended by the WHO, were five times as likely to deliver at a health facility as those who did not make any visits (OR: 5.32; CI: 3.28–8.61). Women’s education was also a significant factor. The predicted odds of women with secondary education (6^th^ grade and above) delivering at a health institution was 40% higher than their counterparts without any formal education (OR: 1.40; CI: 1.05–1.88). As expected, household wealth and urban residence correlated positively with institutional delivery. Compared to the women from poor households, those from wealthy households were twice as likely to have an institutional delivery (OR: 2.29; CI: 1.64–3.19). Urban women were nearly three times more likely to deliver at a health facility than rural women (OR: 2.93; CI: 2.23–3.85). Geographically, women from Terai had 35% higher odds of delivering at a health facility compared to women from the Hill region. Women with one child were nearly three times more likely to deliver at a health facility than women with more than two births (OR: 2.77; CI: 2.06–3.72). There was no difference in institutional delivery between women who had two children and those who had more than two children. Finally, women’s access and exposure to the news media (newspapers/magazines, radio, and television) mattered; the odds of institutional delivery increased by 10% for every one unit increase in the index of news media exposure (OR: 1.10; CI: 1.01–1.20).

**Table 3:** Odds ratio predicting likelihood of women delivering at an institution in Nepal, 2011.

	Model 1—Full Sample (N = 3997): Predicting institutional delivery = 1 and home delivery = 0		
Predictor variables	Odds Ratio	95% CL	
**Age at interview**	1.01	0.98	1.03
**Age at marriage** (20+ = 1; else = 0)	1.22	0.94	1.59
**Number of children** (>2 children = 0)			
One child	2.77***	2.06	3.72
Two children	1.22	0.93	1.61
**Women’s education** (no edu = 0)			
Primary (1-5^th^ grade)	1.05	0.79	1.41
Secondary+ (6^th^ grade & more)	1.40*	1.05	1.88
**Husband’s education** (no edu = 0)			
Primary (1-5^th^ grade)	0.98	0.70	1.38
Secondary+ (6^th^ grade & more)	1.06	0.76	1.48
**Wealth Index** (Poor = 0)			
Middle	1.41*	1.05	1.90
Rich	2.29***	1.64	3.19
**Religion** (Hindu = 1; else = 0)	1.08	0.72	1.60
**Urban** (rural = 0)	2.93***	2.23	3.85
**Ecological region** (Hill = 0)			
Mountain	0.77	0.52	1.15
Terai	1.35*	1.03	1.77
**Problem with money**	0.82	0.65	1.04
**Problem with Distance**	0.82	0.63	1.05
**Exposure to news media index**	1.10*	1.01	1.20
**Exposed to health education programs (**yes = 1; no = 0)	0.92	0.73	1.16
**# of antenatal care visits during last pregnancy** (no antenatal care visits = 0)			
1 to 3 visits	2.01**	1.25	3.24
4 or more visits	5.32***	3.28	8.61
**Knows about Cash Incentives program** (yes = 1; no = 0)	2.70***	1.59	4.59
F value for Wald χ^2^	27.64***		
Max-rescaled R^2^	.42		
*C*	.84		
Hosmer and Lemeshow χ^2^ GFI	13.41		

*p < .05

**p< .01

*** p < .001

## Goodness of fit test and sensitivity analysis

The Chi-squares for Hosmer and Lemeshow goodness-of-fit test statistics and the corresponding *p* values computed from the chi-square distribution with 8 degrees of freedom were not significant (χ^2^ = 13.41; p = .10), indicating that the model had a good fit because the observed and model-predicted frequencies were not statistically different. Additionally, to assess misclassification of binary outcome variables, I evaluated the Receiver Operating Characteristic (ROC) curve derived by plotting true response (Sensitivity) and false response (1-Specificity), an overall assessment of accuracy [52]. Theoretically, the value of concordance index (*c* statistic) or the area under the curve ranges from 0.5 to 1, where 0.5 suggests that the model randomly predicts the response and 1 indicates that the model perfectly discriminates the response. The closer the value of *c* to 1, the higher the level of correct classification. In our model, the *c* statistics was 0.84, indicating that the model had a strong level of discrimination [52]. Additionally, to assess misclassification of binary outcome variables, sensitivity analyses were conducted. The results of sensitivity, specificity, false positive, and false negatives showed that the correct classification rates at three different probability cut-off points (cut-off points 0.40, 0.50 and 0.60 chosen to reflect the prevalence of institutional delivery in Nepal), ranged from 75% to 76%.

## Study limitations

This study has several limitations. First, Nepal began implementing the SDIP nationwide in 2005. The data for this analysis come from the survey conducted in 2011 in which the women who had given births in the previous five years were asked about the place of birth. By 2011, nearly 90% of the respondents indicated that they knew about the cash incentive policy, but many had delivered at home. It is possible that some of the women who delivered at home heard about the policy after their last delivery. There was no way to verify when they heard about the cash incentives and if more of them would have delivered at a health facility had they heard about the program before delivery. This is an important limitation of the current study. An analysis of the next wave of NDHS data will help address this limitation. Second, delivery decisions could depend on the quality of health facilities and service providers that the women were able to access. Some health facilities may be better equipped and staffed to attend to deliveries, with a better reputation among the users than other facilities. This study does not capture the variations in the quality of the facility or women’s previous interaction with staff in these facilities. Third, family members play a critical role in facilitating institutional delivery [53]. For example, as time for birth nears, women need supportive spouses, family members, relatives and neighbors to accompany them to a health facility. This study, however, could not control for this factor. Fourth, it is not clear if women knew about the change in rules about the SDIP in 2007. Pregnant women may have received an inconsistent message about the cash incentive which may have affected their decisions to deliver at home or institution. For example, some women with two children may have opted to deliver at home because under the 2005 rule they would not qualify for the cash incentive although this rule was changed in 2007. Fifth, we do not know to what extent the SDIP influenced the quality of delivery services. The SDIP also transferred money to health care providers who attended to deliveries and to small institutions in remote areas of Nepal if they serviced deliveries. As there were financial incentives for attending to each delivery, it is possible that the institutions made changes. Perhaps, the institutions improved the quality of services to accommodate deliveries. The rate of institutional deliveries may have increased as the quality of health facility improved and women acted on this knowledge and expectations of better hospitals instead of increased knowledge about the SDIP. This is a threat to internal validity of this study. Sixth, the binary, independent variable, was created using one question in the survey that asked “*Does a woman get a cash incentive if she delivers her baby at a government health facility*? This is an imperfect measure of knowledge about the SDIP. Women who requested a private ward for their delivery and would be ineligible for the cash transfer and they could have answered no to their knowledge about the SDIP. In the current sample, there was no way to know how many women requested a private ward for their delivery. Among those who delivered at the health facility and did not receive any cash, however, 8% did not know about the SDIP. As expected, everyone who delivered at the health facility and received cash knew about the SDIP. It is not clear, however, if they learned about the program after they came to deliver at the health facility or if they chose institutional delivery due to their knowledge about the SDIP.

Finally, and most importantly, the relationship between women’s awareness about the cash program and their likelihood of institutional delivery is correlational at best and not causal due to the cross-sectional nature of the data. The endogeneity of the independent variable could not be ruled out. There may also be a selection bias. It is possible that the women who were aware of the cash program were different from those who were not aware of the program in some observable and unobservable variables. To test for the selection bias, the dependent variable, institutional delivery was replaced with four new variables outside of the current study. These included if the respondents belonged to women’s group coded as 1 (40% belonged to women’s group), if they used solid fuel (e.g., coal, wood, agricultural crop waste) over cleaner fuels such as electricity or natural gas (83% used solid fuel), if they smoked tobacco products (12% smoked tobacco products), and if they owned property—house or land (10% owned property). These four variables were regressed on knowledge about the cash program along with all other control variables listed in Table 1. The results showed that the knowledge about the cash program was not associated with belonging to women’s group (OR = 0.99; CI: 0.69–1.14), use of solid fuel (OR = 0.83; CI: 0.42–1.61), use of tobacco products (OR = 1.33; CI: 0.73–2.40) and owning property (OR = 1.06; CI: 0.51–2.19) controlling for the covariates. In other words, it is safe to assume that the women who knew and did not know about the SDIP were similar in their membership to women’s group, use of solid fuel for cooking, use of tobacco products and their property ownership status. They were, however, statistically different in their institutional delivery behavior. The results of these tests provide greater confidence in the presence of a relationship between awareness about the cash program and institutional delivery observed in this study. They do not, however, infer a causal relationship between awareness of the cash program and increase in institutional deliveries.

## Discussion

High maternal and child mortality is a concern for developing countries like Nepal. Much progress has been made in building health institutions and training health workers. Additionally, Nepal now offers a cash incentive to all women who come to deliver in public or private health facilities, unless they request a private ward. This study examined the impact of the SDIP on institutional delivery. What follows is a discussion of key findings.

First, it is important to note that almost 90% of Nepalese women who had given births in the past five years knew about the cash incentive program. Of the women who did not receive any cash, perhaps they requested a private ward for their delivery, 92% knew about the about the SDIP. Nepal needs to pay more attention to the remaining 10% of the women who are still unaware of the policy. The government could disseminate the SDIP policy through all community health and social work professionals, so that all women, irrespective of their pregnancy status learn about the policy. Also, the SDIP has become more inclusive since its inception in 2005 with the removal of the restriction of “up to two children” and expansion of the program to private health institutions. Future studies could examine the proportion of women who knew about the revised policy and if more of them chose to deliver at the health institution due to the policy changes.

Second, the rate of institutional delivery has doubled in Nepal between 2006 and 2011 from 20% to 39% [54]. This study shows that some of this increase in institutional delivery is associated with the SDIP. Women who knew about the program were almost three times more likely to deliver at an institution than their counterparts who did not know about the program, controlling for other factors. This result is consistent with findings from other countries in South Asia. In West Bengal, India the JSY program doubled the likelihood of institutional delivery [28]. In several other states of India where the JSY cash payment was more generous, it also increased institutional delivery among poor and less educated women [27, 55, 56]. This study provides further evidence and reassurance that the CCT programs are associated with increased institutional delivery in different countries and context. In line with SLT, as more women have positive experience delivering at health institutions and endorse institutional delivery, it is reasonable to assume that their family and friends will imitate and model these behaviors until institutional delivery becomes the norm as in the developed countries [44, 45].

Third, in Nepal, even with the SDIP, nearly 61% of the women delivered at home presenting a considerable risk for maternal and child health. Of the women who knew about the SDIP, 58% delivered at home (Table 1). These women knew about the SDIP but chose to deliver at home. This shows that a complex set of factors determine the delivery decisions and that knowledge about the cash program is not sufficient for institutional delivery. One explanation for home delivery even among women who knew about the cash incentives could be social incompatibility of services provided. To create local demand for institutional delivery, the services have to be socially compatible with traditional practices [36]. For example, while giving birth in the comfort of their own home, women are traditionally attended to by female family members or a female birth attendant from their community. Given this tradition, women may feel uncomfortable being attended to by a male care provider at the health facility. While women are transitioning from home to institutional delivery, to the extent possible, healthcare institutions could simulate existing traditions by ensuring sufficient female staff attending each institutional delivery.

Another possibility for low institutional delivery among women who knew about the SDIP could be due to the low access and quality of delivery care provision in health facilities in Nepal. Studies from other low-and-middle-income countries have suggested that access to quality health care continues to be a problem in developing countries. While low-income countries have expanded their health care infrastructures, several studies have pointed out that the quality of care provided or the standards of care that the patients receive are often low and need improvement [57–59]. A systematic review of 80 studies from low-and-middle income countries assessing the quality of healthcare provision concluded that both public or private sectors scored low on infrastructure, clinical competence, and practice[60]. Poor and less educated patients may receive poorer quality of care due to systematic discrimination of these patients [59]. While there were no studies examining the quality of delivery care in Nepal, one study assessed the quality of antenatal care provided. The authors examined if the patient received seven WHO recommended components of services during their antenatal care visit: blood pressure measurement; urine tests for detecting bacteriuria and proteinuria; blood tests for syphilis and anemia; and provision of iron supplementation, intestinal parasite drugs, tetanus toxoid injections and health education [61]. Their study concluded that socioeconomically disadvantaged women—less educated and poor—were less likely to receive the recommended care [61]. This study recommended targeting poor and less educated women in Nepal and making sure that they receive better quality services.

Additionally, women need to see the relative advantage of institutional delivery in terms of cost and benefit and the degree to which it is perceived as being beneficial to home delivery (e.g., safer environment for delivery, lower maternal and child death) [34–36]. Money and distance to a health facility were not significant in this study, perhaps because the questions were more general and did not specify pregnancy or birth. The questions asked if money and distance were barriers to treatment when they were sick. Studies that have examined the effect of cost and distance to health facility on the use of health services during pregnancy and birth have found them to be significant. If it takes longer than an hour to travel to the birthing facility, it deters women from choosing that option [46, 53]. In one study, 28% of those who delivered at home indicated that they did so to avoid the cost of institutional delivery [46]. It is possible that the delivery care costs more than the incentive offered. For example, studies evaluating similar cash incentive programs in India found that some of the women had out-of-pocket expenditure to cover the cost of prescription drugs, consumables, and transportation charges [62, 63]. As the majority of women in Nepal continue to deliver at home, researchers and policymakers should test if an upward adjustment to the reimbursement amount will increase the rate of institutional delivery.

Fourth, consistent with previous studies, antenatal care use, women’s education, exposure to news media, household wealth status, number of children, and urban residence play an important role in institutional delivery even under the cash incentive policy [47–49, 54]. Among these variables, antenatal care use is the strongest predictor of institutional delivery. While cash incentive is a worthy policy intervention, health and social workers must continue to push for women’s increased access to antenatal care. There is evidence that institutional delivery increases among women who engage in birth preparation [48, 53]. A study from a remote mountainous district of Nepal noted that the non-institutional delivery was associated with the lack of delivery preparation and limited access to health services—including cost, distance to the facility, and lack of trust toward the health care system and its staff [46]. Social workers could encourage pregnant, mainly rural, low-income, less educated women to attend antenatal care visits and think about preparing for birth.

Finally, it was interesting to note that younger women in their first pregnancy were more likely to seek institutional delivery. Even in remote mountainous districts of Nepal, the women were significantly more likely to choose a health institution for their first birth than for their subsequent births [46]. For subsequent deliveries, they preferred home. We need to understand the driving forces for this behavior. For example, it is not clear, to what extent women choose home delivery for subsequent births due to the cost of first delivery, social incompatibility or negative experience at the institution, or other factors? The quality of health facilities, women’s previous interaction and trust toward service providers may determine their future use of these services. Also, some health facilities are poorly equipped and staffed to attend deliveries than other facilities. Some facilities may have earned a better reputation among the users than other facilities. We need to understand these and other factors that drive the choice of delivery behavior.

### Conclusion and implications

About 61% of Nepali women continue to deliver at home, and this is a risk factor for maternal and child death. To increase institutional delivery, Nepal has introduced the SDIP. This program is universal; all women in Nepal, unless they request private wards, are eligible to receive a cash incentive. The knowledge of the SDIP was associated with nearly three-fold increase in institutional delivery. Nearly 90% of the women who had delivered in the past five years knew about the SDIP. The 10% of women who still do not know about the program are at a much higher risk for choosing home delivery and developing maternal and child health complications than other women who know about the SDIP. Health and social work professionals should educate these women about the SDIP so that they can make informed delivery decisions. Also, they should find ways to bring the 15% of the women who skipped antenatal care into the health care system as that will increase the likelihood of institutional delivery.
